# The Remarkable Legacy of Theodor O. Diener (1921–2023): Preeminent Plant Pathologist and the Discoverer of Viroids

**DOI:** 10.3390/v15091895

**Published:** 2023-09-08

**Authors:** Robert A. Owens, Ahmed Hadidi

**Affiliations:** U.S. Department of Agriculture, Agricultural Research Service, Beltsville, MD 20705, USA; owensj301@hotmail.com

Theodor (“Ted”) Otto Diener, the discoverer of viroids, died on 28 March 2023 at his home in Beltsville, Maryland, USA. He was 102 years old.

During the early and middle portions of the 20th century, many diseases of unknown etiology with virus-like symptoms, but for which no virions could be found, were described. The infectious nature of one such disease, potato spindle tuber, was demonstrated by Schultz and Folsom in the 1920s [1], and in 1967 Ted Diener and William Raymer obtained evidence that the agent of this disease was not a virus but rather a very small, protein-free RNA [2]. Four years later, Ted conclusively demonstrated in two landmark articles [3,4] that the causal agent was an infectious, autonomously replicating and very small single-stranded RNA. He proposed the term “viroid” to differentiate this unconventional agent and other RNAs with similar properties from the multitude of conventional viruses [4].

The discovery of viroids in 1971 represents the third major extension in the history of the biosphere, following the earlier discoveries of “subvisible” microorganisms by the Dutch microbiologist Antonie van Leeuwenhoek in 1675 and of the “submicroscopic” viruses by the Russian botanist Dmitri I. Ivanovsky in 1892 [5]. The discovery of the potato spindle tuber viroid (PSTVd) also revealed the existence of previously unsuspected regulatory pathways in plants. Over the succeeding 50+ years, almost 40 additional species of viroids have been isolated from a wide variety of vegetable and ornamental crops including fruit trees, palms, and grapevine [6]. The International Committee on Virus Taxonomy (ICTV) recognized the discovery of viroids by establishing a new order of subviral agents that currently includes two families: *Pospiviroidae* with five genera and thirty-five species, and *Avsunviroidae* with three genera and only five species [7].



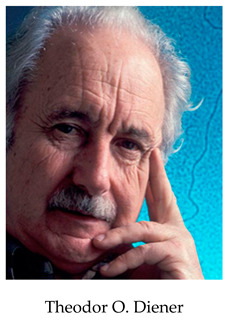



The Noble Prize-winning physicist Erwin Schrodinger (1887–1961) reminds researchers that “the task is not to see what has never been seen before, but to think what has never been thought before about what you see every day” [8]. The opening pages of Ted’s autobiography, “Of humans, humanoids, and viroids” [9], recounts how early-life experiences created the frame of mind essential for his later scientific accomplishments. Ted was born on 28 February 1921 in Zurich, Switzerland, the only child of Theodor and Hedwig (Bauman) Diener. Neither his father, a postal worker, nor his mother, an accountant, shared his childhood enthusiasm for small living things, and gradually an internal rebelliousness brought on by the hypocrisy of the adults around him matured into profound doubt and full-blown skepticism.

After spending World War II as an airplane mechanic for the Swiss Air Force, Ted completed his doctorate in biology at the Swiss Federal Institute of Technology (ETH), Zurich, Switzerland, in 1948. Another notable alumnus of the same institution is Albert Einstein. Ted then emigrated to the United States in 1949, where he accepted a position at Washington State College (now Washington State University) in Prosser, working with viruses that infect fruit trees. After becoming a naturalized U.S. citizen, he moved to Beltsville, Maryland, in 1959 as a founding member of the Agricultural Research Service’s Plant Virology Laboratory, one of that organization’s post Sputnik “pioneering laboratories”.

When Ted Diener arrived in Beltsville, potato spindle tuber disease had been studied for more than 50 years. A lengthy article about the infectious nature of this disease was published in 1923 [1]. Although the causal agent was assumed to be a conventional plant virus, all efforts to purify this hypothetical virus were unsuccessful. Two other Beltsville researchers, William B. Raymer, and Muriel J. O’Brien had developed a relatively rapid bioassay for the causal agent using Rutgers tomato rather than potato (its natural host) [10], but the infectious agent present in extracts from diseased plants would not sediment into a pellet when subjected to ultracentrifugal forces sufficient to sediment all known viruses. It was at this point that Ted Diener joined the effort to characterize this unusual pathogen.

His 2003 article “Discovering viroids-a personal perspective” [11] describes how he combined then State-of-the-Art technology (i.e., ultracentrifugation and polyacrylamide gel electrophoresis) with tomato bioassays to demonstrate that the causative agent of this disease could not be (i) a plant virus whose coat protein is either non-functional or yields only very unstable virions, (ii) a satellite RNA whose replication is totally dependent on the presence of a helper virus, or (iii) a collection of small RNAs that together could represent a viral genome of conventional size. Exceptional claims are often said to require exceptional evidence, and Ted’s 1971 announcement of the discovery of the potato spindle tuber viroid [3,4] is a masterpiece.

Many virologists and molecular biologists outside the plant virology community were slow to accept the existence of viroid(s). One reason for this reluctance was the dependence upon a systemic bioassay rather than conventional biochemical and biophysical techniques to detect their presence. The viroid concept also contradicted a widely-held (though mostly unspoken) belief that only a much larger genome (~10^6^ Daltons) could contain sufficient genetic information for autonomous replication. For those choosing to adopt a “wait and see” attitude, additional evidence for the existence of viroids soon appeared. Similar agents were isolated from two other crop species (i.e., citrus exocortis viroid [12] and chrysanthemum stunt viroid [13]), and just seven years later Heinz Sänger and colleagues, in Germany, published the complete nucleotide sequence of PSTVd [14]. Notably, PSTVd was the first eukaryotic pathogen to have its genome completely sequenced.

The two of us joined Ted’s laboratory just as this pioneering phase of viroid research was drawing to a close. The fact that a low molecular weight RNA could replicate autonomously in a variety of host species apparently uninfected by another agent had a number of important implications for virology, molecular biology, and genetics, and we set about investigating the molecular processes responsible for viroid replication, movement, and pathogenicity. A steady stream of visiting scientists attracted by Ted’s growing reputation—John Randles (University of Adelaide, Australia), Junji Hashimoto (Tsukuba Science City, Japan), Jorge Galindo (Centro de Fitopatologia, Texcoco, Mexico), Thierry Candresse (INRA, Bordeaux, France), Po Tien (Chinese Academy of Sciences, Beijing, China), Teruo Sano (Hirosaki University, Japan), and others—created an exciting and highly productive atmosphere for research. 

The Agricultural Research Service is a mission-oriented organization that emphasizes the value of fundamental research to solve practical problems affecting American farmers. The same molecular tools and techniques used in fundamental studies of viroid molecular biology proved to be just what was needed to develop a series of rapid and sensitive diagnostic tests for viroid infection [15,16]. Thanks to long-term collaborations with Luis Salazar and other members of the International Potato Center (Lima, Peru) as well as Chester Sutula, founder of Agdia, Inc. (Elkhart, IN, USA) and his group, these testing methods rapidly led to the virtual elimination of PSTVd from potato breeding programs in North America and Europe.

The accelerating pace of molecular studies involving viroids produced an ever-increasing number of complete viroid nucleotide sequences, thereby allowing Ted to turn his attention to the question of viroid origin. In 1989, he proposed that viroids and certain viroid-like satellite RNAs may be “living fossils” from a pre-cellular RNA world [17]. Consistent with such a scenario, initial phylogenetic analyses indicated a monophyletic origin for viroids, selected plant satellite RNAs, and the viroid-like domain of human hepatitis delta virus RNA [18]. The small size and circularity of viroids would have enhanced the probability of survival under the primitive error-prone conditions faced by self-replicating RNA systems and assured complete replication without the need for initiation or termination signals.

Although leaving the apparent absence of viroids from prokaryotic algae (ancestors of modern higher plants) unexplained, this RNA world hypothesis is still widely accepted. Earlier this year, however, the outlines of an alternative hypothesis began to emerge. Two independent metagenomic analyses of environmental samples indicate that fungi provide an evolutionary hub for RNA viruses and viroid-like elements [19,20]. Furthermore, evidence has been presented for the transmission of the apple scar skin viroid from plant to plant-associated fungi under natural conditions [21].

Ted was also intensely interested in the possibility that animals or humans might also harbor viroids. When he discovered PSTVd in 1971, two neurodegenerative diseases—one known as “kuru” that was associated with cannibalism in New Guinea and a second disease of sheep and goats known as “scrapie”—seemed possible candidates. Experimental studies had shown the scrapie agent to be highly resistant to UV inactivation, indicating that its genome (presumably either DNA or RNA) must be quite small. In 1972, when Ted raised the possibility of a viroid etiology for scrapie [22], Stanley B. Prusiner was well advanced in his efforts to purify the scrapie agent and had given the name “prions” (proteinaceous infectious particles) to these partially purified preparations [23]. The two investigators decided to collaborate on studies comparing properties of PSTVd with those of the scrapie agent.

The viroid provided an important control for these studies, allowing Stanley Prusiner and Ted to show that not only were prions not viruses, but they were also not viroids. The viroid was inactivated by procedures that modify RNA, whereas the infectivity of the prion was preserved. Conversely, the prion was inactivated by procedures that modify protein, but the infectivity of the viroid was not affected. The results of this collaboration were published in 1982 [24], and later presented at a symposium entitled “Subviral pathogens of plants and animals: viroids and prions” sponsored by the Rockefeller Foundation and held in Bellagio, Italy, in the summer of 1983. In 1997, Stanley Prusiner was awarded the Nobel Prize in Physiology or Medicine “for his discovery of Prions, -a new biological principle of infection”.

Ted Diener’s many contributions to biological science have been widely recognized. Elected Fellow of the American Phytopathological Society in 1973, he was the 1976 recipient of its Ruth Allen Award for “an outstanding, innovative research contribution that has changed, or has the potential to change, the direction of research in any field of plant pathology”. In 1975, Ted shared the Alexander von Humboldt Award with Joseph Semancik of the University of California, Riverside; two years later, he was elected to the U.S. National Academy of Sciences. In 1980, Ted was elected member of the Leopoldina (German Academy of Sciences). In 1987, he was awarded both the Wolf Prize in Agriculture (Israel) and the National Medal of Science (USA). A member of the Agricultural Research Service’s Science Hall of Fame, Ted joined the University of Maryland as a Distinguished Professor in 1989 following his retirement from federal service. In 2000, the American Phytopathological Society declared his discovery of the viroid to be one of the six most important discoveries of the millennium to involve pathogens [25].

Ted Diener’s first marriage, to Shirley Baumann, ended in divorce. In 1968, he married Sybil Fox, who died in 2012. He is survived by three sons from his first marriage, Michael, Theodore, and Robert; five grandchildren; and three great-grandchildren. In his later years, and always with a twinkle in his eye, Ted Diener would occasionally remind us of the long-established Swiss “custom” of providing troublesome family members, such as his paternal great-great grandfather, with a one-way ticket to America. Ted Diener’s decision to leave Switzerland for the USA, in contrast, marked the beginning of a long and highly creative life of remarkable scientific accomplishment. We were fortunate indeed to have begun our careers in viroid research as members of his laboratory.
